# Vitamin C treatment attenuates hemorrhagic shock related multi-organ injuries through the induction of heme oxygenase-1

**DOI:** 10.1186/1472-6882-14-442

**Published:** 2014-11-12

**Authors:** Bing Zhao, Jian Fei, Ying Chen, Yi-Lin Ying, Li Ma, Xiao-Qin Song, Jie Huang, Er-Zhen Chen, En-Qiang Mao

**Affiliations:** Department of Emergency Intensive Care Unit, Ruijin Hospital, Shanghai Jiao Tong University School of Medicine, 197 Ruijin 2nd Road, Shanghai, 200025 China; Department of Surgery, Ruijin Hospital, Shanghai Jiao Tong University School of Medicine, Shanghai, 200025 China; Department of Emergency Intensive Care Unit, the Third People’s Hospital, Shanghai Jiao Tong University School of Medicine, Shanghai, 200025 China

## Abstract

**Background:**

Vitamin C (VitC) has recently been shown to exert beneficial effects, including protecting organ function and inhibiting inflammation, in various critical care conditions, but the specific mechanism remains unclear. Induction of heme oxygenase (HO)-1, a heat shock protein, has been shown to prevent organ injuries in hemorrhagic shock (HS) but the relationship between VitC and HO-1 are still ill-defined so far. Here we conducted a systemic *in vivo* study to investigate if VitC promoted HO-1 expression in multiple organs, and then tested if the HO-1 induction property of VitC was related to its organ protection and anti-inflammatory effect.

**Methods:**

Firstly, to determine the HO-1 induction property of VitC, the HO-1 level were measured in tissues including kidney, liver and lung of the normal and HS model of Sprague–Dawley (SD) rats after VitC treatment (100 mg/kg body weight). Secondly, to testify if VitC prevented HS related organ injuries via inducing HO-1, the HS model of rats were separately pre- and post-treated with VitC, and some of them also received Zinc protoporphyrin (Znpp), a specific HO-1 inhibitor. The HO-1 activity in tissues was tested; the organ injuries (as judged by histological changes in tissues and the biochemical indicators level in serum) and inflammatory response in tissues (as judged by the level of pro-inflammatory cytokines Tumor necrosis factor-α and Interleukin-6 ) were analyzed.

**Results:**

The HO-1 mRNA and protein level in kidney, liver, and lung were highly induced by VitC treatement under normal and HS conditions. The HO-1 activity in tissues was enhanced by both VitC pre- and post-treatment, which was shown to improve the organ injuries and inhibit the inflammatory response in the HS model of rats. Of note, the beneficial effects of VitC were abolished after HO-1 activity was blocked by Znpp.

**Conclusions:**

VitC led to a profound induction of HO-1 in multiple organs including the kidney, liver and lung, and this property might be responsible for the organ protection and inflammation inhibitory effects of both pre- and post-treatment with VitC in HS.

## Background

Hemorrhagic shock (HS) with trauma is the leading cause of death for individuals aged 5 to 44 years [1]. Recent clinical studies show that multiple organ failure (MOF) remains common in patients with HS during the first two days after admittance into the ICU, and the persistence of MOF has been shown to be associated with a high mortality rate [2]. To date, the prevention of MOF in the early pathological process of HS is of utmost importance. Pro-inflammatory cytokines, such as tumor necrosis factor (TNF)-α and interleukin (IL)-6, have been found to be important in the development of MOF in patients with HS [3]. Our previous study also showed that down-regulation of TNF-α and IL-6 led to improvement in HS-related organ injuries [4].

HO-1, also known as heat shock protein 32, is the rate-limiting enzyme in heme catabolism [5, 6] with the anti-oxidant and anti-apoptosis effect. Recently, the exogenous induction of heme oxygenase (HO)-1 has received an increasing amount of attention due to its anti-inflammatory potential. Over-expression of HO-1 exerts anti-inflammatory effects not only through the enzymatic degradation of pro-inflammatory free heme [7], but also through the production of the anti-inflammatory compounds bilirubin and carbon monoxide (CO) [8]. It has been demonstrated that exogenous induction of HO-1 exhibits protective effects on a number of organs, including the kidneys [9], liver [10], and lungs [11] in the animal model of HS.

Parenteral supplementation of vitamin C (VitC) was recently shown to exert inflammation inhibitory and organ protective effects in several different kinds of critical conditions, such as sepsis [12], cardiac arrest [13] and burn injury [14]. Recently, Schreiber et al. [15, 16] showed resuscitation with VitC decreased the level of IL-6 in serum and lung tissue in a swine model of HS. However, the specific mechanism through which VitC exerts these beneficial effects has not been fully elucidated.

To the best of our knowledge, the studies on the relationship between VitC and HO-1 are sparse, and the results seem contradictory. An early report [17] showed that VitC significantly induced HO-1 expression in gastric epithelial AGS and endothelial KATO IIIT, which might correlate with gastro-protection properties. A study of leukemia [18] showed that VitC potentiated the therapeutic efficacy of As^3+^ by enhancing the expression of HO-1. Recently, VitC pretreatment was shown to induce HO-1 expression in neurons and glial cells and attenuate methamphetamine-induced reactive oxygen species (ROS) production and neurotoxicity [19]. In contrast, VitC was shown to inhibit the dopamine mediated HO-1 induction in human umbilical vein endothelial cells in a dose-dependent pattern [20], and partly antagonized resveratrol mediated HO-1 induction in cultured hepatocytes [21]. Therefore, to determine the relationship between VitC and HO-1 may help further elucidate the protective mechnism of VitC.

For the first time, we conducted a systemic *in vivo* study to determine if VitC induced HO-1 expression in the kidneys, liver, and lungs which are relatively vulnerable in the pathological process of HS. In order to mimic more clinical situations, such as prophylactic therapy in cases with a high risk of massive blood loss and the rapid resuscitation for the trauma-hemorrhagic shock patients, VitC was delivered before and after the establishment of HS model, and the organ injuries and pro-inflammatory cytokines level were evaluated. To explore the mediating role of HO-1, some VitC treated HS model of rats further received the specific inhibitor of HO-1 activity, Zinc protoporphyrin (Znpp).

## Methods

### Animals

This study was carried out in strict accordance with the guidelines for the care and use of laboratory animals established by the Animal Use and Care Committee of the Shanghai Committee on Animal Care. Animal surgical procedures were approved by the Institutional Animal Care and Use Committee (IACUC) at Shanghai Jiao Tong University, Shanghai, China (Permit Number: SCXK [shanghai] 2008–0016). Adult male Outbred Sprague–Dawley (SD) rats (body weight = 250 ± 10 g) were purchased from Shanghai Laboratory Animal Center (Chinese Academy of Science, China) and housed under specific pathogen-free conditions. The rats were provided rodent chow and tap water ad libitum with a 12 hours/12 hours light/dark cycle.

### HS model

The establishment of the HS model was performed according to the method described by Kana Umeda et al. [22] with slight modifications. In brief, the rats were anesthetized with intraperitoneal sodium pentobarbital (50 mg/kg body weight). The left and right femoral artery were dissected using aseptic techniques and cannulated with a heparinized polyethylene tube. Catheters were inserted into the left femoral artery to monitor blood pressure (Powerlab 15 T, ADInstrument, Australia) and into the right femoral artery to induce hemorrhage. After the baseline blood pressure was measured, hemorrhage was initiated by bleeding into a heparinized syringe (10 units/mL) over a period of 15 minutes to obtain a mean arterial pressure of 30 mmHg. This blood pressure level (30 ± 5 mmHg) was maintained for 1 hour by withdrawing more blood or reinfusing the shed blood (average bleeding volume: 6 ± 0.5 ml). As the HS operation was completed, the animals were resuscitated for 15 minutes by returning all of the shed blood and then administering a volume of Ringer’s solution equal to the volume of the shed blood. The Sham rats were cannulated but were not subjected to hemorrhage. The rats were allowed to breathe spontaneously throughout the experiment. To maintain the body temperature within the physiological range, all of the procedures were performed over a heating pad, and the rectal body temperature was continuously monitored. Electrocardiography was also continuously measured.

### Experimental design

To examine the effect of VitC treatment on HO-1 expression in the liver, kidney, and lung under normal condition, rats were intraperitoneally injected with VitC (100 mg/kg body weight, Sigma, St. Louis, MO, USA), as described previously [23, 24]. The VitC was dissolved in normal saline (NS, 10 mg/ml) and filtered through a 0.45-μm filter (Millipore, Billerica, MA, USA) immediately before use. Then, 2 to 2.5 mL of the VitC solution was administered to each rat (the exact volume depended on the weight of the animal). The control rats received the same volume of NS. Then, at 2, 6, 12, 24 hours after the VitC or NS administration, the tissue samples including kidneys, liver, and lungs were collected for further analyses. To further investigate the effect of VitC on tissue HO-1 expression after HS, the rats underwent HS operation (HS group) and then immediately were intraperitoneally treated with VitC (100 mg/kg body weight, HSV_-post_ group). Rats in the Sham and HS groups were given NS as a control. The tissue samples were collected at 2, 6, 12, 24 hours after sham or HS operation for further analyses.

To investigate if pre- and post- treatment protected against organ injuries in HS via the induction of HO-1, the HS model of rats (HS group) were separately pre-treated with VitC at 6 hours before HS operation (HSV_-pre_ group) and post-treated with VitC (100 mg/kg body weight) immediately after the HS operation (HSV_-post_ group). Some rats in HSV_-pre_ and HSV_-post_ group further received ZnPP (3 mg/kg body weight, Frontier) at 1 hour before HS operation (HSV_-pre_Z group and HSV_-pre_Z group), as previously described [25]. The rats undergone sham (Sham group) received VitC (100 mg/kg body weight) at 6 hours before the sham operation (ShamV group). Some rats in ShamV group further received Znpp at 1 hour before the sham operation (ShamVZ group). The rats in the Sham and HS groups were given NS as a control. At 0 and 12 hours after sham or HS operation, the tissue samples and serum were collected for further analyses.

### Real-time PCR

After the samples were homogenized in liquid nitrogen, the TRIzol Reagent (Invitrogen, USA) was added to the tissue samples, and 2 μg of total RNA was extracted. The reverse transcription reaction was conducted in a mixture containing random primers, RevertAid Reverse Transcriptase, RNase inhibitor, and dNTP (all of the reagents were obtained from Thermo Scientific Inc., Lithuania, EU) to generate the single-strand cDNA. The PCR reaction mixture was prepared using SYBR Premix Ex Taq (Takara Bio Inc, Shiga, Japan) with the specific upstream and downstream primers for mRNA analysis. The thermal cycling conditions were 10 seconds at 95°C for the initial denaturation step followed by 40 cycles of 95°C for 5 seconds and 60°C for 20 seconds on a 7500 real-time PCR system (7500, ABI, Foster, USA). The relative mRNA expression levels were normalized to β-actin and calculated using the ΔΔCt method. The HO-1 mRNA level was expressed relative to the control group, and the mRNA levels of TNF-α and IL-6 were expressed relative to the Sham group. The sequences of the upstream and downstream primers for HO-1, TNF-α, IL-6, and β-actin are shown in Table 1.

**Table 1:** **Sequences of the upstream and downstream primers used in this study**

Gene	Primer sequences (5′→3′)
TNF-α	upper CCCAATCTGTGTCCTTCTAACT
	lower CACTACTTCAGCGTCTCGTGT
IL-6	upper CAAAGCCAGAGTCATTCAAGC
	lower GGTCCTTAGCCACTCCTTCTGT
HO-1	upper ACCCCACCAAGTTCAAACAG
	lower GAGCAGGAAGGCGGTCTTAG
β-actin	upper GCGCTCGTCGTCGACAACGG
	lower GTGTGGTGCCAAATCTTCTCC

### Western-blot analysis

Equal amounts of protein extract (40 μg) from the kidney, liver, and lung were loaded onto a 10% resolving gel for electrophoresis. The proteins were transblotted onto a Hypond polyvinylidene fluoride membrane (0.45 μm, Millipore, Temecula, CA, USA). The membranes were blocked by incubation in phosphate-buffered saline containing 0.1% Tween 20 and 5% nonfat milk for 60 minutes at room temperature. The blot was immune-probed with HO-1 primary antibody (1:1000 dilution, Abcam, Cambridge, MA, USA) and an anti-β-actin antibody (1:1000 dilution, Santa Cruz Biotechnology, Dallas, TX, USA) in succession overnight at 4°C. The blots were then incubated with an HRP-conjugated secondary antibody for 1 hour at room temperature and then reacted with an enhanced chemiluminescence substrate (Pierce, Rockford, IL, USA). The resulting chemiluminescence was recorded with an imaging system (Imagequant LAS 400, GE, USA). To quantify the protein expression, the enhanced chemiluminescence signals were digitized using the Photoshop CS6 software (Adobe, USA).

### Immunohistochemistry

All of the protocols followed the guidelines of Histostain-Plus Kits (Invitrogen, Frederick, MD, USA). Briefly, the tissue samples were fixed in 10% neutral buffered formalin, embedded in paraffin, and sectioned into 4 to 6 μm-thick sections. After the antigen was retrieved in a citrate buffer (0.01 M, pH 6.0) and subjected to heat treatment using a microwave, the nonspecific binding sites were blocked with 10% non-immune goat serum for 30 minutes. The slides were then incubated at 4°C overnight with a rabbit polyclonal HO-1 antibody (1:400 dilution, Abcam, USA). The slides were further incubated with biotinylated secondary antibody for 1 hour. Normal mouse serum was used as a control for nonspecific staining. The images were collected with a Zeiss microscope M1 (Jena, Germany).

### HO-1 activity assay

The tissue samples were perfused in situ with NS until the venous effluent became clear and then removed for the preparation of microsomes. Based on the methods described by Liu et al. [26], the HO-1 activity was measured through the spectrophotometric determination of the formation of bilirubin according to the manufacturer’s instructions (Genmed Scientifics, Arlington, MA, USA). The amount of bilirubin in the tissue lysate was determined by the difference in the absorbances at 464 and 530 nm using an extinction coefficient of 40 mM^-1^ cm^-1^. The enzymatic activity was expressed as the picomoles of bilirubin formed per milligram of cell protein per hour (pmol mg^-1^ h^-1^).

### Histological study

The tissue samples were fixed in 10% neutral buffered formalin, embedded in paraffin, and sectioned into 4 to 6 μm thick sections. After deparaffinisation and dehydration, the sections were stained with hematoxylin and eosin for microscopic examination. The histological changes observed in the slides were blindly scored, as described previously [27, 28]. Briefly, the severity of renal tubular injury was scored by estimating the percentage of tubules in the cortex or the outer medulla that exhibited epithelial necrosis or had luminal necrotic debris, tubular dilation, and hemorrhage: 0, none; 1, <5%; 2, 5 to 25%; 3, 25 to 75%; and 4, >75%. The severity of liver injury was scored as follows: 0, minimal or no evidence of injury; 1, mild injury consisting of cytoplasmic vacuolation and focal nuclear pyknosis; 2, moderate to severe injury with extensive nuclear pyknosis, cytoplasmic hypereosinophilia, and loss of intercellular borders; and 3, severe necrosis with disintegration of the hepatic cords, hemorrhage, and neutrophil infiltration. The severity of lung injury was scored as follows: 0, no evidence of injury; 1, mild injury; 2, moderate injury; and 3, severe injury with pulmonary edema, interstitial inflammatory cell infiltration, and hemorrhage. All of the evaluations were performed on five fields per section and five sections per organ.

### Analysis of serum biochemical indicators

As soon as collected, the arterial blood samples were immediately centrifuged at 3,000 g for 15 minutes to obtain the serum. The serum levels of alanine aminotransferase (ALT), aspartate aminotransferase (AST), blood urea nitrogen (BUN), creatinine (Cre), lactic dehydrogenase (LDH), and lactate were measured using an automatic biochemical analyzer (UniCel DxC 800, Beckman Coulter, USA).

### Enzyme linked immunosorbent assay

Protein levels of TNF-α and IL-6 in tissues were quantified using an enzyme linked immunosorbent assay (ELISA) kit (Mosaic ELISA system, R&D systems, Minneapolis, MN) according to the manufacturer’s protocol. Samples were measured in duplicate. Readings from each sample were normalized for protein concentration.

### Statistical analysis

The statistical analysis was performed using Prism 4 software (GraphPad Software, San Diego, CA, USA). All the data are expressed as the mean ± SEM and compared using the unpaired Student’s t-test and a one-way analysis of variance followed by Turkey’s test. The differences with a probability value of *p <* 0.05 were considered significant.

## Results

### HO-1 mRNA and protein expression in tissues were highly induced under normal condition by VitC

**Figure 1:**
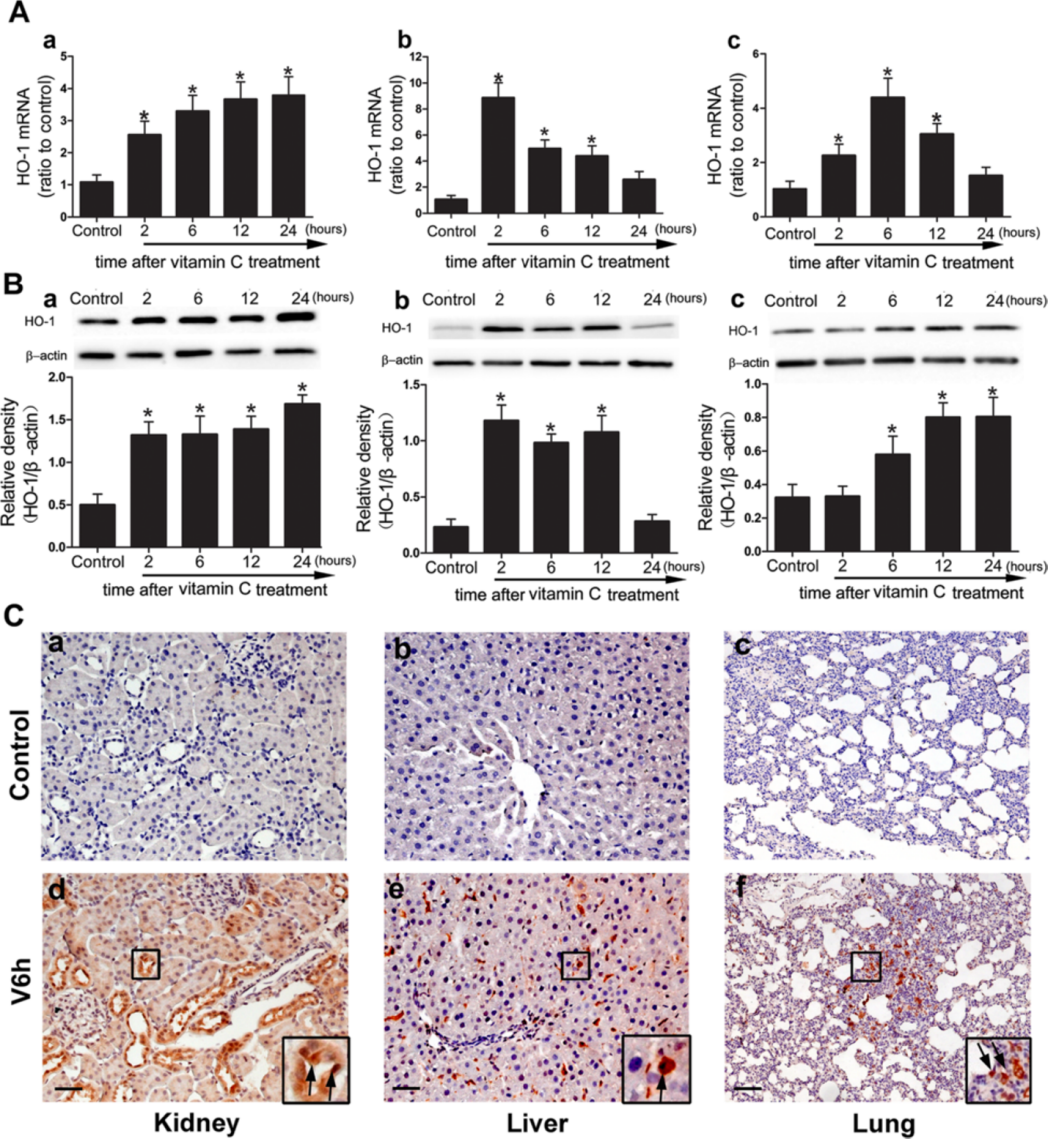
**HO-1 mRNA and protein expression in tissues was highly induced under normal condition by VitC. A)** HO-1 mRNA in the kidney **(a)**, liver **(b)**, and lung **(c)** are expressed relative to the level measured in the control animals. **B)** HO-1 protein expression in the kidney **(a)**, liver **(b)**, and lung **(c)** were detected using Western-blot. Each gel is representative of six independent experiments. Densitometric analysis was used to calculate the normalized protein ratio (HO-1 to β-actin). **C)** distribution of HO-1 protein in the kidney (**a**, **d**, scale bar: 200 μm), liver (**b**, **e**, scale bar: 100 μm), and lung (**c**, **f**, scale bar: 200 μm) were demonstrated by immunohistochemistry. The arrowheads indicate the HO-1-positive cells. Each image is representative of six independent experiments (n = 6). Tissue samples were collected at 2, 6, 12 and 24 hours after VitC treatment. Data represent the mean ± SEM. *p < 0.05 vs. the control. Control: normal saline-treated rats. V6h: the rats treated with VitC for 6 hrs.

Compared to NS, the VitC treatment induced a significant and gradual increase of HO-1 mRNA and protein level in the kidney, which reached a plateau at 24 hours after VitC treatment (Figure 1Aa and 1Ba). The HO-1 mRNA level in the liver was markedly increased and reached a maximum at 6 hours and was then gradually decreased to around the base level at 24 hours after VitC treatment (Figure 1Ab), and the protein level peaked at 2 hours and was decreased to the base level at 24 hours after VitC treatment (Figure 1Bb). The HO-1 mRNA level in the lung was gradually increased and reached a maximum at 6 hours and was decreased to the base level at 24 hours after VitC treatemtn (Figure 1Ac), and the protein level was significantly increase at 6 hours and peaked at 24 hours after VitC treatment (Figure 1Bc). The immunohistochemistry analysis showed the distribution of the HO-1 protein at 6 hours after VitC treatment. The positive staining of the HO-1 protein was mainly observed in the renal tubular epithelial cells (Figure 1Cd), the hepatic parenchymal cells (Figures 1Ce) and the lung epithelial cells (Figure 1Cf), which are the targeted cells in ischemic/reperfusion (I/R) injury of the liver and kidney [29, 30] as well as acute lung injury [31]. There was little positive staining in the kidney (Figure 1Ca), liver (Figure 1Cb), and lung (Figure 1Cc) of the Control rats.

### The increased HO-1 mRNA and protein expression in tissues after HS was enhanced by VitC

**Figure 2:**
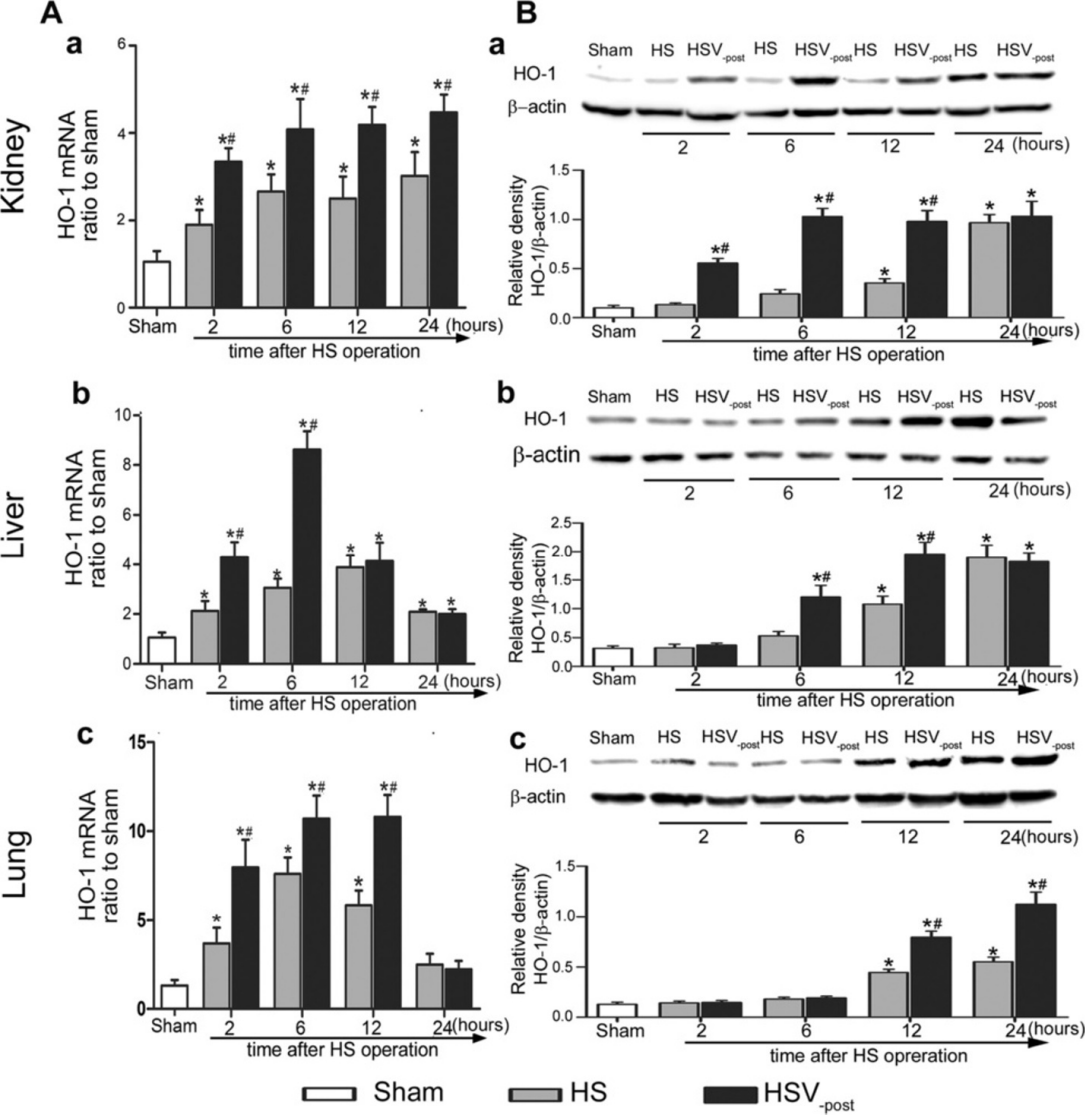
**The increased HO-1 mRNA and protein expression in tissues after HS were enhanced by VitC. A)** HO-1 mRNA in the kidney **(a)**, liver **(b)**, and lung **(c)** are expressed relative to the level measured in Sham group. **B)** HO-1 protein expression in the kidney **(a)**, liver **(b)**, and lung **(c)** were detected using Western-blot. Each gel is representative of six independent experiments. Densitometric analysis was used to calculate the normalized protein ratio (HO-1 to β-actin). Tissue samples were collected at 2, 6, 12 and 24 hours after HS operation. Data represents the mean ± SEM. *p < 0.05 vs. the Sham; ^#^ *p* < 0.05 vs. the HS at the same time point. Sham: the rats which underwent sham operation and received normal saline (NS). HS: the rats which underwent HS operation and received NS. HSV_-post_: the rats which underwent HS operation and then received VitC immediately.

Compared to Sham group, the HO-1 mRNA level in kidney of HS group was significantly increased at 2, 6, 12 and 24 hours after HS operation, which was enhanced by VitC post-treatment (Figure 2Aa), while the HO-1 protein level was significantly increased at 12 and 24 hours after HS operation compared to Sham group, which was significantly enhanced by VitC post-treatment at 2, 6 and 12 hours after HS operation (Figure 2Ba).

The HO-1 mRNA level in liver of HS group, significantly increased at 2, 6, 12 and 24 hours after HS operation, was greatly enhanced VitC post-treatment at 2 and 6 hours after HS operation (Figure 2Ab), while the HO-1 protein level, significantly increased at 12 and 24 hours after HS operation, was enhanced by VitC post-treatment at 6 and 12 hours after HS operation (Figure 2Bb).

The HO-1 mRNA level (Figure 2Ac) in lung was significantly increased at 2, 6 and 12 hours, while the protein (Figure 2Bc) level reached the plateau at 12 and 24 hours after HS operation, both of which was greatly up-regulated by VitC.

### HO-1 activity in tissues was highly enhanced by both VitC pre- and post-treatment

**Figure 3:**
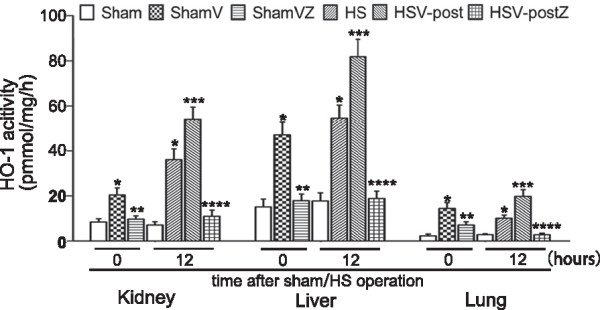
**HO-1 activity in tissues was enhanced by both VitC pre- and post-treatment.** Tissue samples were separately removed at 0 and 12 hours after sham or HS operation for HO-1 activity assay. *p < 0.05 vs. the Sham at the same time point; **p < 0.05 vs. the ShamV; ^***^ *p <* 0.05 vs. the HS; ^****^ *p <* 0.05 vs. the HSV_-post_. Data represents the mean ± SEM, n = 6. Sham: the rats which received NS and underwent sham operation; ShamV: the rats which received VitC and underwent sham operation; ShamVZ: some ShamV rats further received ZnPP; HS: the rats which received NS and underwent HS operation; HSV_-post_: the rats which underwent HS operation and then received VitC immediately; HSV_-post_Z: some HSV_-post_ rats further received ZnPP.

As the HO-1 activity reflects the enzymatic function, it is essential to investigate the effect of VitC pre- and post-treatment on HO-1 activity in tissues. As the Figure 3 indicated, compared to Sham group, the HO-1 activity in tissues of ShamV group was significantly increased, which was significantly reduced after the addition of Znpp. The HO-1 activity in tissues was significantly increased at 12 hours after HS operation. The HO-1 activity in tissues of HSV-_post_ group, significantly higher than the level of HS group, was decreased after the addition of Znpp.

### Both VitC pre- and post-treatment relieved histological damages through HO-1 induction

**Figure 4:**
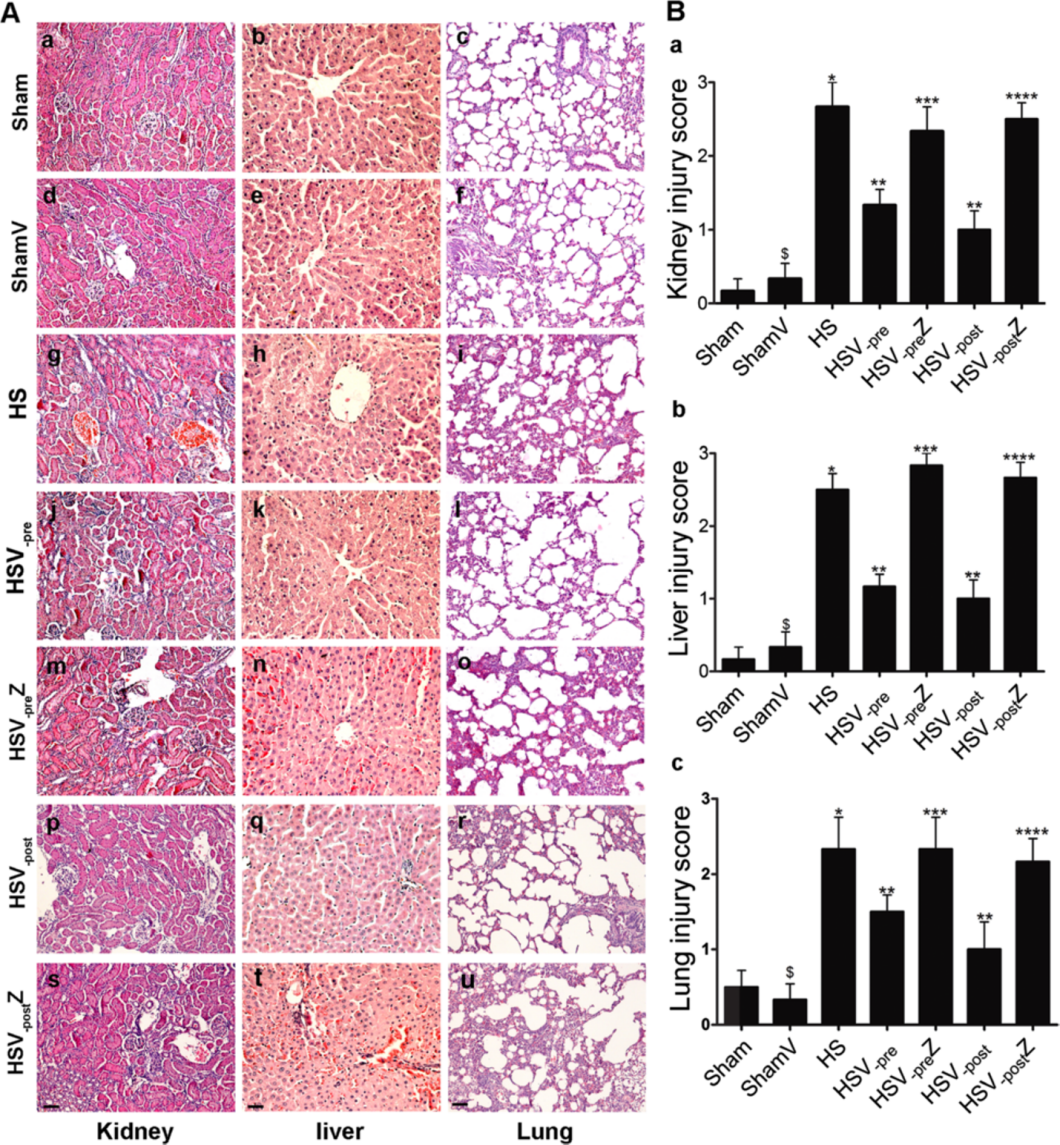
**Both VitC pre- and post-treatment relieved histological damages through HO-1 induction. A)** representative light microscopy images of the kidney (**a**, **d**, **g**, **j**, **m**, **p**, **s**, scale bar: 200 μm), liver (**b**, **e**, **h**, **k**, **n**, **q**, **t**, scale bar: 100 μm), and lung (**c**, **f**, **I**, **l**, **o**, **r**, **u**, scale bar: 200 μm), which were stained with hematoxylin and eosin. **B)** histological damage score of the kidney **(a)**, liver **(b)**, and lung **(c)**. Tissue samples were collected at 12 hours after the HS operation. Data represents the mean ± SEM, n = 6. *p < 0.05 vs. the Sham; **p < 0.05 vs. the HS; ***p < 0.05 vs. the HSV_-pre_; ****p < 0.05 vs. the HSV_-post_; ^$^ *p* > 0.05 vs. the Sham. Sham: the rats which received NS and underwent sham operation. ShamV: the rats which received VitC and underwent sham operation. HS: the rats which underwent HS operation and received NS. HSV_-pre_: the rats which received VitC at 6 hours before HS operation. HSV_-pre_Z: some HSV_-pre_ rats further received ZnPP. HSV_-post:_ the rats which underwent HS operation and received VitC immediately. HSV_-post_Z: some HSV_-post_ rats further received ZnPP.

At 12 hours after HS operation, obvious histological damage was observed in the kidneys, liver, and lungs through hematoxylin and eosin staining. These damages included epithelial necrosis, tubular dilation, and hemorrhage in the kidneys (Figure 4Ag), extensive nuclear pyknosis, hepatic cell necrosis, and hemorrhaging in the liver (Figure 4Ah), and pulmonary edema, interstitial inflammatory cell infiltration, and hemorrhaging in the lungs (Figure 4Ai). Both VitC pre- (Figures 4Aj-l) and post-treatment (Figures 4A-r) improved the histological damages in HS group, and the beneficial effects of VitC were attenuated by the addition of ZnPP (Figures 4Am-o and 4As-u). All of these histological changes were quantitatively evaluated using injury scores (Figure 4Ba-c).

### Both VitC pre- and post-treatment decreased the serum level of biochemical indicators through HO-1 induction

**Figure 5:**
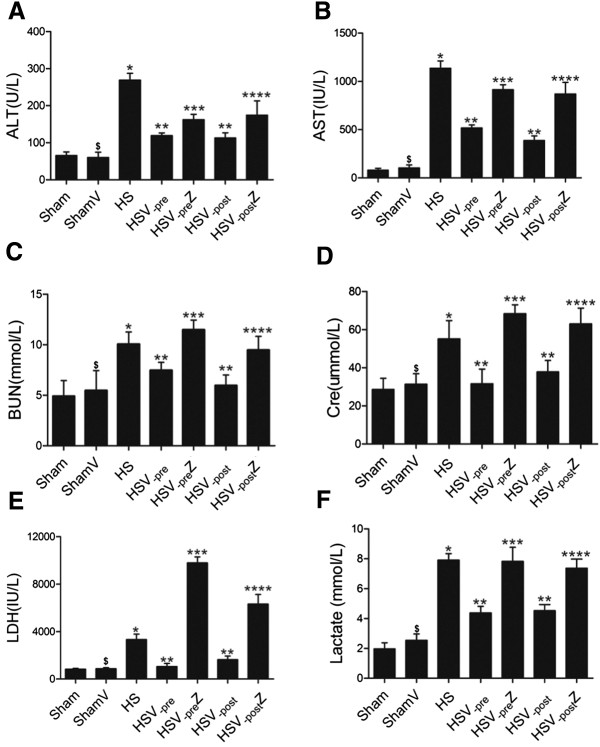
**Both VitC pre- and post-treatment decreased the serum level of biochemical indicators through HO-1 induction.** Serum level of biochemical indicators including ALT **(A)**, AST **(B)**, BUN **(C)**, Cre **(D)**, LDH **(E)**, and lactate **(F)** were tested. Serum was collected at 12 hours after HS operation. Data represents the mean ± SEM, n = 6. *p < 0.05 vs. the Sham; **p < 0.05 compared to the HS; ***p < 0.05 compared to the HSV; ****p < 0.05 compared to the HSV_-post_; ^$^ *p* > 0.05 compared to the Sham. Sham: the rats which received NS and underwent sham operation. ShamV: the rats which received VitC and underwent sham operation. HS: the rats which received NS and underwent HS operation. HSV_-pre_: the rats which received VitC at 6 hours before HS operation. HSV_-pre_Z: some HSV_-pre_ rats further received ZnPP. HSV_-post:_ the rats which underwent HS operation and received VitC immediately. HSV_-post_Z: some HSV_-post_ rats further received ZnPP.

At 12 hours after HS operation, the serum was obtained and the level of biochemical indicator including ALT (Figure 5A), AST (Figure 5B), BUN (Figure 5C), Cre (Figure 5D), LDH (Figure 5E) and lactate (Figure 5F) were detected. All of the biochemical indicators were increased in the HS group compared to the Sham group. Both pre- and post-treatment with VitC significantly reduced the levels of all of the indicators, which became pronounced again after the addition of ZnPP.

### Both VitC pre- and post-treatment decreased proinflammatory cytokine levels in tissues through HO-1 induction

**Figure 6:**
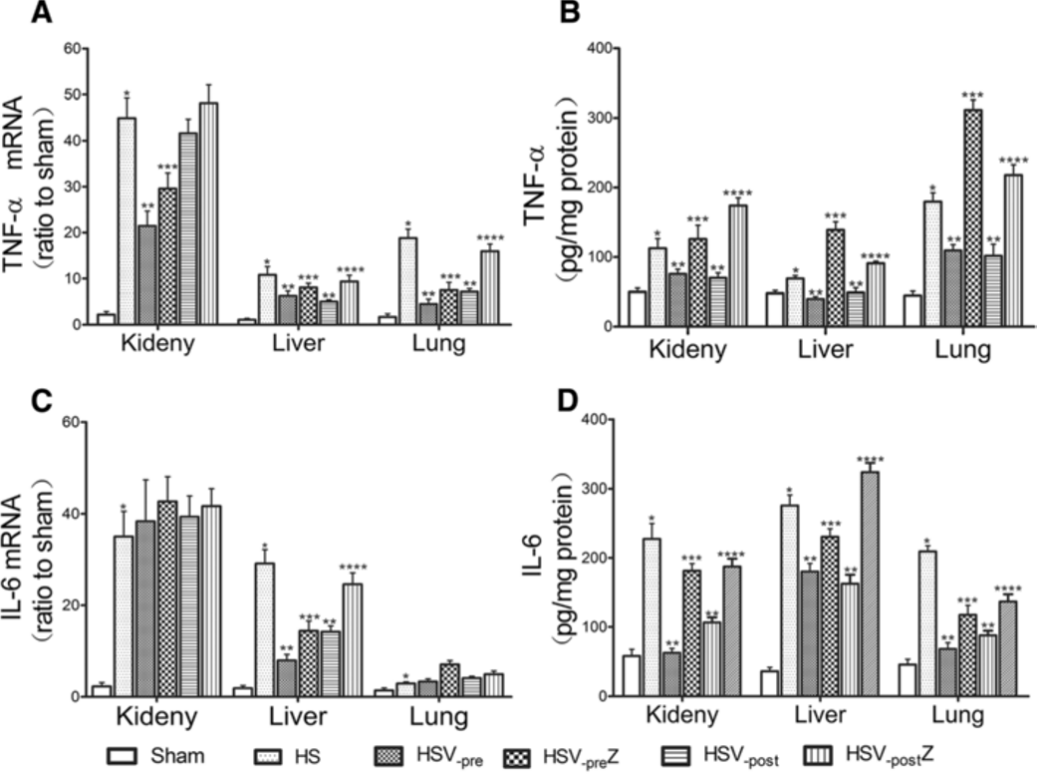
**Both VitC pre- and post-treatment decreased the tissues level of proinflammatory cytokines through HO-1 induction.** The mRNA and protein levels of the proinflammatory cytokines TNF-α **(A, B)** and IL-6 **(C, D)** in tissues collected at 12 hours after HS operation were tested. The data represents the mean ± SEM, n = 6. *p < 0.05 vs. the Sham; **p < 0.05 vs. the HS; ***p < 0.05 vs. the HSV_-pre_; ****p < 0.05 vs. the HSV_-post_. Sham: the rats which received NS and underwent sham operation. ShamV: the rats which received VitC and underwent sham operation. HS: the rats which received NS and underwent HS operation. HSV_-pre_: the rats which received VitC at 6 hours before HS operation. HSV_-pre_Z: some HSV_-pre_ rats further received ZnPP. HSV_-post:_ the rats which underwent HS operation and received VitC immediately. HSV_-post_Z: some HSV_-post_ rats further received ZnPP.

As the HS related organ injuries were mainly ascribed to proinflammatory cytokines [3], the levels of the proinflammatory cytokines TNF-α and IL-6 in the kidneys, liver and lungs were tested at 12 hours after HS operation. The TNF-α mRNA level in liver and lung (Figure 6A) and the IL-6 mRNA level of liver (Figure 6C) elevated in HS group compared to the Sham group, which were relieved by both VitC pre- and post-treatment. This down-regulating effect of VitC was abolished after the HO-1 activity was blocked by the addition of ZnPP. The protein level of TNF-α (Figure 6B) and IL-6 (Figure 6D) in the kidneys, liver, and lungs exhibited a trend similar to that of mRNA levels mentioned above after each treatment.

## Discussion

In this study, we demonstrated that VitC treatment induced HO-1 expression in the kidneys, liver, and lungs under normal condition and enhanced HO-1 expression after HS. Both pre- and post-treatment of VitC led to a marked improvement in organ injuries and inflammatory response. We also demonstrated that administration of ZnPP inhibited HO-1 activity and negated the beneficial effects of VitC treatment. These findings provide more detailed evidences of the mechanism through which VitC exerts a protective effect on HS.

HS and subsequent resuscitation is regarded as a systemic I/R process that causes oxidative injuries of multiple vulnerable organs including the liver [32], lung [33] and kidney [34]. Pre-exposure of these organs to temporal sublethal stress, known as “organ preconditioning”, has been shown to increase the tolerance of the organ to I/R injuries. Several methods for “organ preconditioning” have been reported, such as brief ischemia followed by reperfusion [35], whole-body hyperthermia [36], and the induction of heat shock protein [37], such as HO-1, which is also recognized as heat shock protein 32 [38]. Our study showed the tissues level of HO-1 mRNA (Figure 1A) and protein (Figure 1B) were greatly induced by VitC in normal rats. Furthermore, the HO-1 activity in tissues (Figure 3), which revealed the enzymatic function, were significantly induced by VitC before initiation of hemorrhage. These data confirmed the induction property of VitC and suggested VitC pre-treatment can be considered as an efficient method of “organ preconditioning”.

However, the preconditioning situations make up a relatively small portion of clinical situations. The majority of HS patients are trauma-hemorrhagic patients who are in need of rapid resuscitation. Therefore, it is necessary to investigate the effects of VitC on HO-1 when administrated after HS. Hsu et al. [39] reported resuscitation with estrogen protected cardiac function via enhancing HO-1 expression after trauma-hemorrhagic shock. Consistent with their study, we showed a general trend that VitC post-treatment enhanced HO-1 mRNA (Figure 2A) and protein level (Figure 2B) and HO-1 activity (Figure 3) after HS. This phenomenon reconfirmed the HO-1 induction property of VitC and suggested post-treatment with VitC might exert protective effect via enhancement of HO-1 after HS. Additionally, we showed a discrepancy of HO-1 mRNA and protein in the three organs, and this phenomenon may be attributed to the difference of HO-1 mRNA and protein half-life [40, 41] as well as its tissue specific expression pattern [11].

However, pharmacological VitC is deemed as either a pro-oxidant or an anti-oxidant depending on its concentration which may lead to different consequences [42]. For example, VitC, at the dose of 30 mg/kg, relieved hepatic injuries in a rat model of hepatic ischemia/reperfusion, but aggravated the hepatic injuries at the doses of 1000 mg/kg [43]. As a result, it is possible that the dose of VitC used currently (100 mg/kg) might induce HO-1 expression as its pro-oxidant property and exert potential adverse effect. We showed VitC treatment did not cause obvious organ injuries in Sham rats according to the analyses of the histological changes (Figures 4Ad-f) and the serum levels of various biochemical indictors (Figure 5). Therefore, it is reckoned that the dose of VitC used in our study was adequate to induce HO-1 expression due to its pro-oxidant effect without disturbing normal organ physiology and function. Similar to our findings, VitC has been shown to kill tumor cells through the formation of ROS but impart no adverse effects on normal cells *in vitro* [44].

At 12 hours after HS operation, obvious organ histological injuries were observed (Figure 4), accompanying with the increased serum level of biochemical indicators (Figure 5) which are the markers of organ function (ALT, AST, BUN and Cre) [45] and the indicator of HS severity (LDH and lactate) [46, 47]. The level of proinflammatory cytokines TNF-α and IL-6 in tissue were also increased at the same time (Figure 6). Both pre- and post-treatment with VitC was shown to lead to the marked improvement of organ injuries and a decrease of inflammatory cytokine levels. The benefits of VitC were abolished by ZnPP, a HO-1 specific inhibitor, which was shown to down-regulate the enhanced HO-1 activity following pre- or post-treatment with VitC (Figure 3). Therefore, HO-1 is speculated to play an important role in mediating the protective effect of VitC. Specifically, the benefit of VitC pre-treatment might be related to “organ preconditioning” by pre-induction of HO-1, and VitC post-treatment exerted its beneficial effects by further enhancing the expression of HO-1 induced by HS per se.

## Conclusions

The present systemic study demonstrated that VitC treatment induced HO-1 expression in the kidneys, liver, and lungs *in vivo*. Both VitC pre- and post-treatment improved the HS related organ injuries and the level of pro-inflammatory cytokines via the induction of HO-1. Our data further elucidates the mechanism of VitC for preventing systemic injuries induced by HS.
